# Supplementary material for: Dynamic modeling of mortality risk factors in Ebola virus disease using logistic regression on unbalanced panel data from a randomized controlled trial in the Democratic Republic of Congo
Source: PLOS Glob Public Health. 2025 Jul 11;5(7):e0004901. doi: 10.1371/journal.pgph.0004901 (PMC12250521; doi:10.1371/journal.pgph.0004901)
Supplement: Attachment — Submitted filename: Response to Reviewers.pdf [file pgph.0004901.s005.pdf]

Manuscript Title: Dynamic Modeling of Mortality Risk Factors in Ebola Virus Disease Using Logistic Regression on Unbalanced Panel Data from a Randomized Controlled Trial in the Democratic Republic of Congo

Manuscript ID: PGPH-D-24-02895R1

Dear Dr. Goyal,

We thank you and the reviewers for the thoughtful feedback and the opportunity to revise our manuscript. We have addressed all comments carefully and believe the manuscript has been strengthened as a result. Below, we provide a point-by-point response to each comment and outline the corresponding changes made in the revised version. All changes are visible in the file titled "Revised Manuscript with Track Changes."

Reviewer #1

Comment 1: Keywords, Authors should either use "EVD" or "Ebola Virus Disease" but not "Ebola Virus Disease (EVD)" within the Keywords.

Response: We have revised the keywords for consistency. We now use "Ebola Virus Disease" without including the abbreviation "EVD" in parentheses.
Change made on page 1, Keywords section.

Comment 2: Equation formatting. All equations involving fractions in the manuscript should be written in the form A = B/C (vertical fraction), and not (A = B)/C.

Response: All equations with fractions have been reformatted using vertical fraction notation in accordance with the reviewer's suggestion. Changes made in the Methods section.

Comment 3: Limitations and Future Direction

a. Limitations and Future directions should be part of the conclusion. Authors should summarize them and add them to the ending section of the conclusion, just before the references.

b. Limitations should be stated first before suggestions for future considerations.

c. Suggestions for future considerations should be stated in points just before the references.

Response: We have revised the Conclusion section to include a clearly structured Limitations and Future Directions subsection at the end of the conclusion, in line with the reviewer's recommendation: Limitations are stated first, Future considerations are listed as bullet points.
Change made in the Conclusion section

Reviewer #2

Comment: Consistency in trial name (PALM001-RCT vs PALM-RCT). There are some occurrences of both PALM001-RCT and PALM-RCT. Please review this for consistency.

Response: We have reviewed the entire manuscript and now refer consistently to the trial as “PALM-RCT” throughout the text. All instances of "PALM001-RCT" have been corrected accordingly.

Reference List: As per journal instructions, we reviewed our reference list to ensure accuracy and completeness. We confirm that no retracted articles have been cited. Minor updates and reordering were made to maintain consistency after editing the text. These changes are tracked in the "Revised Manuscript with Track Changes."

Please find attached: Response to Reviewers (this document), Revised Manuscript with Track Changes, Final Clean Manuscript

We thank the editor and reviewers for their time and helpful suggestions. We hope the revised manuscript meets the publication standards of PLOS Global Public Health.

Sincerely,

Mr. Leader Lawanga
On behalf of all co-authors
