# Supplementary material for: Dynamic modeling of mortality risk factors in Ebola virus disease using logistic regression on unbalanced panel data from a randomized controlled trial in the Democratic Republic of Congo
Source: PLOS Glob Public Health. 2025 Jul 11;5(7):e0004901. doi: 10.1371/journal.pgph.0004901 (PMC12250521; doi:10.1371/journal.pgph.0004901)
Supplement: Attachment — Submitted filename: Response to Reviewers.docx [file pgph.0004901.s002.docx]

**Response to Reviewers**

**Manuscript ID:** PGPH-D-242895

**Title:** *Dynamic Modeling of Mortality Risk Factors in Ebola Virus Disease Using Logistic Regression on Unbalanced Panel Data from a Randomized Controlled Trial in the Democratic Republic of Congo*

We sincerely thank the reviewers for their constructive and insightful comments, which have helped us to significantly improve the quality of our manuscript. Below, we provide detailed responses to each comment and indicate the corresponding revisions made in the manuscript.

**Reviewer #1**

**Comment 1: Grammar, punctuation, and typos**

*There are some grammatical errors in the manuscript. I think it needs to be polished further, and some typos need to be revised. Further, punctuation marks should be checked throughout the paper, especially after the equations. Authors need to correct them throughout the manuscript. For instance, in line 232, there should be a full stop behind the validation.*

**Response:**
We appreciate the reviewer’s constructive comment. The manuscript has been thoroughly revised for grammar, spelling, punctuation, and clarity. Specifically, punctuation after equations has been corrected, including the addition of a full stop after “validation” on line 232. Formatting has been standardized throughout the document.

**Comment 2: Equation formatting**
*All equations must be well punctuated, centered, and typed well. For instance, equations in lines 214, 216, 218 should be well. The fraction in line 222 must be typed in the form A=D/C.*

**Response:**
Thank you for this observation. The equations have been reformatted for consistency, clarity, and proper alignment. The fraction in line 222 is now presented in the A = D / C format as recommended.

**Comment 3: Motivation in the Introduction**
*Authors should clearly state their motivation for this research in the introduction section.*

**Response:**
We have revised the Introduction to clearly state the motivation for our study. We emphasize the need to address the limitations of static analyses by introducing a dynamic modeling approach using unbalanced panel data to better understand evolving risk factors for EVD mortality.

**Comment 4: Literature review expansion**
*Authors cited only two papers at the review or related work section. Authors should expand the review of literature, compare their current research to the existing literature, and clearly state the innovation in this research in the introduction section.*

**Response:**
We have expanded the literature review to include additional relevant studies (Loubet et al. (2016), Levine et al. (2015), Yango et al. (2024), and Tshomba et al. (2022)). We compare our dynamic modeling approach to previous research relying on static methods and highlight the methodological innovation of applying unbalanced panel data within a logistic regression framework.

**Comment 5: Source of parameter values**
*Authors should indicate the source of the parameter values used for their simulations. If taken from literature, then they should cite the papers in the current study.*

**Response:**
Thank you for this important point. We have clarified in the Methods section that all parameter values used in the analysis were extracted from the PALM-RCT dataset, with no assumptions taken from external sources.

**Comment 6: Indicate limitations**
*Authors should indicate limitations of their research.*

**Response:**
A paragraph discussing the limitations of our study has been added to the Discussion section. This includes the retrospective design, the single dataset origin, and the need for prospective validation of the model.

**Comment 7: Future direction**
*Authors should indicate the future direction of their research.*

**Response:**
We have included a section outlining future research directions. These emphasize the importance of prospective studies and explore the potential integration of dynamic risk assessment models into clinical practice to improve patient management.

**Comment 8: Implications of findings**
*Authors should state the implication of their findings/results.*

**Response:**
The implications of our findings are now more explicitly stated in the Discussion and Conclusion sections. We highlight the potential for improving EVD patient outcomes through personalized risk stratification and timely clinical interventions.

**Comment 9: Figure labels**
*Titles/ Labels of all figures (graphs) should be given.*

**Response:**
All figures have been updated with clear, descriptive titles and axis labels to improve readability and comprehension.

**Reviewer #2**

**Comment 1: Clarify "new predictors"**
*At D0, six significant predictors of mortality were determined, although seven are listed. At Day7, reference is made to five new predictors. Not all of these are entirely new, as two were previously identified at D0. Would suggest rewording the use of “new predictors.”*

**Response:**
Thank you for this valuable observation. We revised the wording to specify that some predictors identified at Day7 were previously observed at Day0 and are therefore better described as “persisting” or “continuing” rather than “new.”

**Comment 2: Line 155 confusing**
*Line 155: Not sure the significance of it. It is confusing.*

**Response:**
We have revised line 155 for clarity. The sentence has been clarified or removed based on its relevance to the surrounding context.

**Comment 3: Line 206 phrasing**
*Shouldn’t read “further variables can be removed” because of the top-down elimination process?*

**Response:**
Thank you for this suggestion. We clarified the methodology section as follows:
We applied a stepwise variable selection process based on a top-down elimination strategy. Initially, all candidate explanatory variables were included in the logistic regression model. The Wald statistic was used at each step to evaluate the statistical significance of each variable, and the variable with the highest p-value exceeding 0.05 was removed. This iterative process continued until all remaining variables contributed significantly (p < 0.05) to the final model.

**Comment 4: Update "PALM" to "PALM-RCT"**
*Line 245: Update PALM to “PALM-RCT”*

**Response:**
Done. All instances have been updated to “PALM-RCT” throughout the manuscript.

**Comment 5 : Age phrasing**
*Line 265: Consider using “age was consistent across the visits…” vs “consistent distribution between 22 and 29 years of age.”*

**Response:**
We have reworded this sentence to: “Age was consistent across the visits with a mean age around 29 years.”

**Comment 6: Standardize terminology**
*AST vs ASAT, ALT vs ALAT, Haemorrhage, Difficulty breathing vs SoB…*

**Response:**
All medical terms and spellings have been standardized throughout the manuscript (ASAT, ALAT, hemorrhage, shortness of breath, etc.).

**Comment 7: Creatinine levels**
*Line 268: Creatine levels remained at 0.6 at D21 and D28.*

**Response:**
This has been corrected and clarified in the Results section.

**Comment 8: Line 283–285: Recalculate percentages**
*For example, 265 deaths of 617 = 43%?*

**Response:**
Thank you for identifying this issue. Percentages have been reviewed and recalculated accurately. The death rate at inclusion has been updated to reflect these corrections.

**Comment 9: Table 2—add sample sizes**
*Please show the number of participants associated with the Biochemical parameters.*

**Response:**

We appreciate this comment. The global sample sizes for each time point (Day 0, Day 7, Day 14, Day 21, and Day 28) are indicated at the top of the respective columns in Table 2. This ensures that the number of participants contributing to each biochemical parameter at each time point is clearly presented..

**Comment 10: Mental state at D0?**
*Was Mental state captured at D0? Would be good to know what its inclusion means.*

**Response:**
Yes, mental state (coma) was assessed at D0 and included as a candidate variable in our analysis. However, coma was not retained as a significant predictor of mortality at D0 after the variable selection process. Interestingly, coma emerged as a significant predictor at later time points (D7 and D14), reflecting its nature as a late clinical sign of disease severity in Ebola Virus Disease. This finding aligns with clinical knowledge that coma often appears at advanced stages of the illness and is associated with poor prognosis.

**Comment 11: GLMM consideration**
*Was Generalized Linear Mixed Model considered for the modeling?*

**Response:**
We considered Generalized Linear Mixed Models (GLMM) but opted for logistic regression due to its interpretability and satisfactory model performance with unbalanced panel data. This choice is now explicitly discussed in the Methods and Discussion sections.

**Comment 12: Consider removing formulas or clarifying them**
*I think we could describe the statistical methods clearly without the formulas.*

**Response:**
In line with this helpful suggestion, we have simplified the presentation of statistical methods and reduced the use of formulas where possible. We supplemented the methods section with clear narrative explanations to improve accessibility for a broad readership.

We trust that these revisions adequately address the reviewers’ comments and have substantially improved the manuscript.

**Sincerely,**

**Leader Lawanga Ontshick and co-authors**
