# Supplementary material for: Dynamic modeling of mortality risk factors in Ebola virus disease using logistic regression on unbalanced panel data from a randomized controlled trial in the Democratic Republic of Congo
Source: PLOS Glob Public Health. 2025 Jul 11;5(7):e0004901. doi: 10.1371/journal.pgph.0004901 (PMC12250521; doi:10.1371/journal.pgph.0004901)
Supplement: Attachment — Submitted filename: The Editor Plos Global Health R1.docx [file pgph.0004901.s003.docx]

The Editor,

PLOS Global Public Health

Reviewer’s Comments (PGPH-D-24-02895R1)

The authors studied the Dynamic Modeling of Mortality Risk Factors in Ebola Virus Disease Using Logistic Regression on Unbalanced Panel Data from a Randomized Controlled Trial in the

Democratic Republic of Congo.

I have checked the revised version of the manuscript according to the comments given, but I still have a few suggestions to be addressed by the authors. These are:

**Comment 1: Keywords**

Authors should either use ‘’EVD’’ or Ebola Virus Disease **but not** Ebola Virus Disease(EVD) within the Keywords.

**Comment 2: Equation formatting**

All equations involving fractions in the manuscript should be written in the form $\mathbf{A=}\frac{\boldsymbol{B}}{\boldsymbol{C}}$, but not $\boldsymbol{A=B}/\boldsymbol{C}$. Authors should please address that.

**Comment 2: Limitations and Future direction**

a. Limitations and Future directions should be part of the conclusion. Authors should summarize them and add them to the ending section of the conclusion, just before the references.

b. Limitations should be stated first before suggestions for future considerations.

c. Suggestions for future considerations should be stated in points just before the references.

**Conclusion:** The manuscript can be accepted **after these minor revisions have been made** to be published in PLOS Global Public Health.

Thank you.
